# Norcantharidin, a clinical used chemotherapeutic agent, acts as a powerful inhibitor by interfering with fibrinogen–integrin α_II__b_β_3_ binding in human platelets

**DOI:** 10.1111/jcmm.13488

**Published:** 2018-01-25

**Authors:** Chih‐Hsuan Hsia, Wan‐Jung Lu, Kuan‐Hung Lin, Duen‐Suey Chou, Pitchairaj Geraldine, Thanasekaran Jayakuma, Nen‐Chung Chang, Joen‐Rong Sheu

**Affiliations:** ^1^ Graduate Institute of Medical Sciences and Department of Pharmacology College of Medicine Taipei Medical University Taipei Taiwan; ^2^ Department of Medical Research Taipei Medical University Hospital Taipei Taiwan; ^3^ Central Laboratory Shin‐Kong Wu Ho‐Su Memorial Hospital Taipei Taiwan; ^4^ Department of Animal Science School of Life Sciences Bharathidasan University Tiruchirappalli Tamil Nadu India; ^5^ Department of Cardiology Taipei Medical University Hospital Taipei Taiwan

**Keywords:** antithrombosis, fibrinogen, integrin α_II__b_β_3_, norcantharidin, platelet aggregation

## Abstract

During platelet activation, fibrinogen binds to its specific platelet receptor, integrin α_II__b_β_3_, thus completing the final common pathway for platelet aggregation. Norcantharidin (NCTD) is a promising anticancer agent in China from medicinal insect blister beetle. In this study, we provided the evidence to demonstrate NCTD (0.1–1.0 μM) possesses very powerful antiplatelet activity in human platelets; nevertheless, it had no effects on surface P‐selectin expression and only slight inhibition on ATP‐release reaction in activated platelets. Moreover, NCTD markedly hindered integrin α_II__b_β_3_ activation by interfering with the binding of FITC‐labelled PAC‐1. It also markedly reduced the number of adherent platelets and the single platelet spreading area on immobilized fibrinogen as well as clot retraction. Additionally, NCTD attenuated phosphorylation of proteins such as integrin β_3_, Src and FAK in platelets spreading on immobilized fibrinogen. These results indicate that NCTD restricts integrin α_II__b_β_3_‐mediated outside‐in signalling in human platelets. Besides, NCTD substantially prolonged the closure time in human whole blood and increased the occlusion time of thrombotic platelet plug formation and prolonged the bleeding time in mice. In conclusion, NCTD has dual activities, it can be a chemotherapeutic agent for cancer treatment, and the other side it possesses powerful antiplatelet activity for treating thromboembolic disorders.

## Introduction

Platelets are anucleate blood cells with crucial roles in thrombosis under both physiological and pathological conditions. They are critical for maintaining the integrity of the vascular system and are the first line of defence against haemorrhage. On encountering a subendothelial matrix exposed by injury to a vessel, platelets adhere, are activated and become adhesive to other platelets, leading to further aggregation 1. During platelet activation, fibrinogen binds to its specific platelet receptor, glycoprotein (GP) IIb/IIIa complex (also known as integrin α_IIb_β_3_), thus completing the final common pathway for platelet aggregation.

Integrin α_IIb_β_3_ is formed through calcium‐dependent association of α_IIb_ and β_3_, an essential step in normal platelet aggregation and endothelial adherence. In resting platelets, integrin α_IIb_β_3_ is normally in a low activation state, unable to interact with fibrinogen. Platelet stimulation with various agonists can induce a conformational change in integrin α_IIb_β_3_, enabling it to bind to its ligands (*i.e*. fibrinogen and von Willebrand factor), resulting in platelet aggregation onset; this process is known as inside‐out signal transduction 2. Meanwhile, the binding of fibrinogen to active high‐affinity integrin α_IIb_β_3_ becomes progressively irreversible, initiating a series of intracellular signalling events, including intracellular calcium mobilization, tyrosine phosphorylation of numerous proteins, activation of phosphoinositide metabolism and cytoskeleton reorganization; this process is often referred to as outside‐in signalling 2. These outside‐in reactions, originating in the integrin α_IIb_β_3_ bound to fibrinogen, are required for maximal secretion, procoagulation and clot retraction 2.

In addition to the regulation of thrombosis and haemostasis, platelets also have a role in tumour cell growth and metastasis. An interplay between platelets and tumour cells contributes to tumour cell growth and subsequently to malignancy progression and survival 3. Platelet α‐granules are sources of a wide range of growth factors and cytokines contributing to tumour metastasis. Following platelet activation, a myriad of angiogenic proteins (*i.e*. vascular endothelial growth factor) and growth factors are released from the platelets into the tumour microenvironment to enhance tumour cell growth. In addition, platelets facilitate tumour cell adhesion to the vascular endothelium and the formation of a protected tumour cell microenvironment 4. Platelets adhere to tumour cells through adhesion receptors, such as integrin α_IIb_β_3_, GP Ib‐IX‐V and P‐selectin 3, 5. Notably, the abundant platelet integrin α_IIb_β_3_ plays a major role in the platelet–tumour cell interaction process 3, 5, 6. Moreover, tumour cells can induce platelet activation through various mechanisms, and their metastatic potential relies on their ability to activate platelets 3. Tumour cells secrete thrombin and ADP, which further amplify platelet activation and recruit platelets to participate in tumour cell‐induced platelet aggregation (TCIPA) and promote tumour cell survival within the circulation 3, 6, 7. TCIPA also up‐regulates the expression of platelet integrin α_IIb_β_3_ and P‐selectin, which bind to mucin‐type GPs on the surface of tumour cells, thereby potentiating platelet–tumour cell interactions 8.

**Figure 1:**
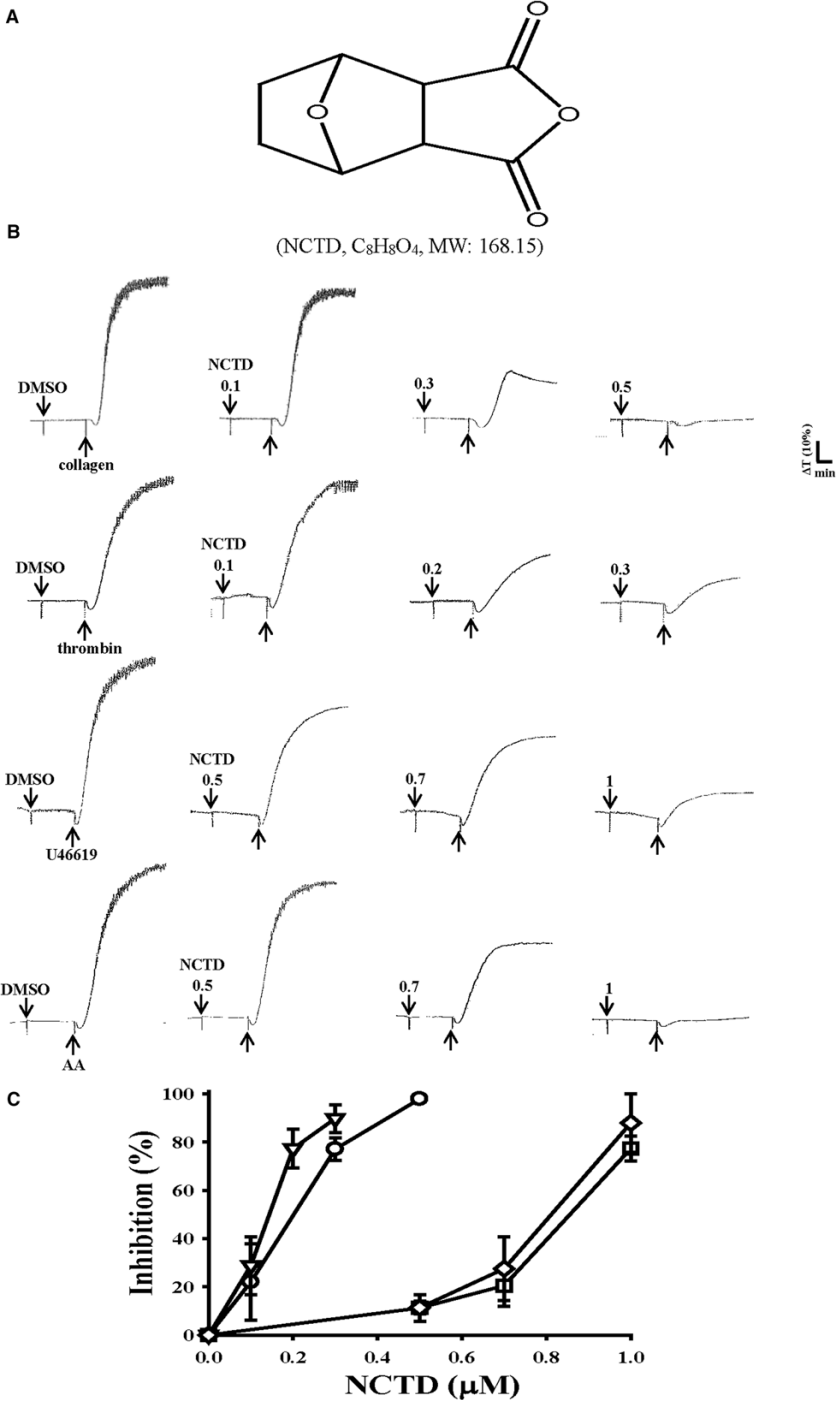
Inhibitory activity of norcantharidin (NCTD) in platelet aggregation stimulated by various agonists in washed human platelets. (**A**) Chemical structure of NCTD. (**B**) Washed human platelets (3.6 × 10^8^ cells/ml) were preincubated with the solvent control (0.1% DMSO) or NCTD (0.1–1.0 μM) and subsequently treated with 1 μg/ml collagen (○), 0.01 U/ml thrombin (▽), 1 μM U46619 (□) and 60 μM arachidonic acid (AA; ♢) to stimulate platelet aggregation. (**C**) Concentration–response curves of NCTD in inhibition of platelet aggregation (%). All data are presented as means ± standard errors of the means (*n *=* *4).

Norcantharidin (NCTD; exo‐7‐oxabicylo‐[2.2.1] heptane‐2,3‐dicarboxylic anhydride) is a demethylated analogue of cantharidin (7‐oxabicyclo‐[2.2.1] heptane‐2,3‐dicarboxylic acid) derived from the dried body of a medicinal insect, the blister beetle (*Mylabris phalerata* Pallas; Fig. 1A) 9. In China, NCTD has been used to treat patients with cancers, such as hepatocellular carcinoma, breast cancer, colon cancer and leukaemia, for many years 10. The antitumour activities of NCTD are multifarious: it can cause apoptosis, inhibit angiogenesis and metastasis in many cell lines and affect multiple pathways controlling cell proliferation 11. In addition, NCTD can repress breast cancer cell adhesion to platelets through down‐regulation of integrin α_2_, an adhesion molecule present on the cancer cell surface 12. Our preliminary findings revealed that NCTD exhibits extremely strong inhibitory activity against the activation of human platelets, thus encouraging us to further examine the characteristics and functional activity of NCTD in platelet activation. This study provides novel evidence that in addition to antitumour activity, NCTD has potent antiplatelet activity through the blockade of fibrinogen–integrin α_IIb_β_3_ binding, which may elicit its antithrombotic and antitumour activities. Therefore, NCTD can be developed into a new class of antiplatelet agents.

## Materials and methods

### Chemicals and reagents

NCTD (99.5%), collagen, luciferin–luciferase, U46619, heparin, prostaglandin E_1_ (PGE_1_), bovine serum albumin (BSA), arachidonic acid (AA), fibrinogen, FITC‐phalloidin and thrombin were purchased from Sigma‐Aldrich (St. Louis, MO, USA). An anti‐integrin β_3_ monoclonal antibody (mAb) and anti‐phospho‐integrin β_3_ (Tyr^759^) polyclonal antibody (pAb) were purchased from Santa Cruz Biotechnology (Santa Cruz, CA, USA). Anti‐phospho‐Src family (Tyr^416^) and anti‐phospho‐FAK (Tyr^397^) mAbs, as well as an anti‐Src family pAb, were purchased from Cell Signaling (Beverly, MA, USA). An anti‐Focal adhesion FAK pAb was obtained from Millipore (Billerica, MA, USA). FITC‐anti‐human CD42P (P‐selectin) and FITC‐anti‐human CD41/CD61 (PAC‐1) mAbs were obtained from BioLegend (San Diego, CA, USA). Protein G Mag Sepharose Xtra Beads were purchased from GE Healthcare (Uppsala, Sweden). A Hybond‐P PVDF membrane, an enhanced chemiluminescence Western blotting detection reagent, horseradish peroxidase (HRP)‐linked donkey antirabbit immunoglobulin G (IgG) and sheep antimouse IgG were purchased from Amersham (Buckinghamshire, UK). The Dade Behring PFA‐100 collagen/ADP (CADP) test cartridge was obtained from Siemens Healthcare (Erlangen, Germany).

### Platelet aggregation

This study was approved by the Institutional Review Board of Taipei Medical University and conformed to the directives of the Declaration of Helsinki. All human volunteers involved in this study provided informed consent. Human platelet suspensions were prepared as described previously 13. Human blood samples were obtained from adult volunteers who had refrained from the use of drugs or other substances that could interfere with the experiment for at least 14 days before collection; the collected blood samples were mixed with an acid–citrate–dextrose solution. After centrifugation, the platelet‐rich plasma was supplemented with 0.5 μM PGE_1_ and 6.4 IU/ml heparin. Tyrode's solution containing 3.5 mg/ml BSA was used to prepare the final suspension of washed human platelets; the final Ca^2+^ concentration in Tyrode's solution was 1 mM. The platelet aggregation test was performed with a lumi‐aggregometer (Payton Associates, Scarborough, ON, Canada) as described previously 13. Various concentrations of NCTD or solvent control (0.1% DMSO) were preincubated with platelet suspensions (3.6 × 10^8^ cells/ml) for 3 min. before adding the agonist (*i.e*. thrombin). The extent of platelet aggregation was calculated as the percentage of light transmission units of the control (without NCTD) after the reaction proceeded for 6 min. For ATP release assay tests, 20 μl of luciferin–luciferase was added 1 min. before adding the agonist; the amount of ATP released was compared with that released by the control.

### Detection of lactate dehydrogenase

Washed platelets (3.6 × 10^8^ cells/ml) were preincubated with 10, 20 and 50 μM NCTD or the solvent control (0.1% DMSO) for 20 min. at 37°C. An aliquot of the supernatant (10 μl) was deposited on a Fuji Dri‐Chem slide LDH‐PIII (Fuji, Tokyo, Japan), and the absorbance wavelength was read at 540 nm on an ultraviolet‐visible spectrophotometer (UV‐160; Shimadzu, Japan). The maximal level of lactate dehydrogenase (LDH) from Triton‐lysed platelets was noted.

### Flow cytometric analysis of surface P‐selectin expression and integrin α_IIb_β_3_ activation in human platelets

Platelet P‐selectin expression and integrin α_IIb_β_3_ activation were detected through flow cytometry. In brief, washed platelets (3.6 × 10^8^ cells/ml) were preincubated with NCTD (0.15 and 0.3 μM) and the FITC‐conjugated anti‐P‐selectin mAb (2 μg/ml) or FITC‐conjugated PAC‐1 mAb (2 μg/ml) for 3 min. and then stimulated by thrombin (0.01 U/ml) for another 5 min. The suspensions were then assayed for fluorescein‐labelled platelets on a flow cytometer (FACScan system; Becton Dickinson, San Jose, CA, USA). Data were collected from 50,000 platelets per experimental group, and the platelets were identified on the basis of their characteristic forward and orthogonal light‐scattering profiles. All experiments were repeated at least four times to ensure reproducibility.

### Confocal microscopic analysis of platelet adhesion and spreading

Here, eight‐chamber glass tissue culture slides were coated with either BSA (100 μg/ml) or fibrinogen (100 μg/ml) at 4°C overnight. After being washed with phosphate‐buffered saline (PBS) two times, the slides were blocked with 1% BSA in PBS for 1 hr and then again washed with PBS two times. Washed platelets (3.0 × 10^8^ cells/ml) preincubated with NCTD (0.15 and 0.3 μM; 5 min. at 37°C) or the solvent control (0.1% DMSO) was allowed to spread on the protein‐coated surfaces at 37°C for 45 min. After the removal of unbound platelets and two washes with PBS, the bound cells were fixed (4% paraformaldehyde), permeabilized (0.1% Triton) and stained with FITC‐labelled phalloidin (10 μM) for 1 hr. All confocal studies were performed with a Leica TCS SP5 microscope equipped with a 63×, 1.40 NA oil immersion objective (Leica, Wetzlar, Germany). Platelet adhesion (cell number) and the platelet surface area (spreading) were determined using NIH ImageJ software (NIH, Bethesda, MD, USA; http://rsbweb.nih.gov/ij/).

### Platelet‐mediated clot retraction

Washed platelets (3.6 × 10^8^ cells/ml) were resuspended in Tyrode's solution containing 2 mg/ml fibrinogen and 1 mM CaCl_2_ and then dispensed in 500‐μl aliquots in glass tubes designed for aggregation 14. NCTD (0.15 and 0.3 μM) or the solvent control (0.1% DMSO) was included in the platelet suspension buffer (3 min., 37°C) prior to clot retraction induction by thrombin (0.01 U/ml) without stirring. The reaction was developed at 37°C in an aggregometer tube and photographed at the indicated times of 15 and 30 min., respectively.

### Immunoblotting

Dishes (6‐cm diameter) were precoated with fibrinogen (100 μg/ml) overnight at room temperature and then blocked with 1% BSA. Washed human platelets (3.6 × 10^8^ cells/ml) were preincubated with NCTD (0.15 and 0.3 μM) or the solvent control (0.1% DMSO) for 3 min. and then added to immobilized fibrinogen dishes for 60 min. at 37°C. The reaction was then stopped, and the platelets were immediately resuspended in 200 μl of lysis buffer. Samples containing 80 μg of protein were separated through 12% SDS gel electrophoresis, and the proteins were electrotransferred to PVDF membranes using a Bio‐Rad semi‐dry transfer unit (Bio‐Rad, Hercules, CA, USA). The blots were then blocked with Tris‐buffered saline in Tween 20 (TBST; 10 mM Tris base, 100 mM NaCl and 0.01% Tween 20) containing 5% BSA for 1 hr and probed with various primary antibodies. The membranes were incubated with HRP‐linked antimouse IgG or antirabbit IgG (diluted 1:3000 in TBST) for 1 hr. An enhanced chemiluminescence system was used to detect immunoreactive bands, and their optical density was quantified using Bio‐profil Biolight (version V2000.01; Vilber Lourmat, Marne‐la‐Vallée, France).

### Immunoprecipitation

In this experiment, dishes (6‐cm diameter) were precoated with fibrinogen (100 μg/ml) overnight at room temperature and then blocked with 1% BSA. Washed human platelets (3.6 × 10^8^ cells/ml) were preincubated with 0.3 μM NCTD or the solvent control (0.1% DMSO) for 3 min. and then allowed to spread on immobilized fibrinogen dishes for 60 min. at 37°C. The platelets were lysed and centrifuged; subsequently, TBS containing Protein G Mag Sepharose Xtra beads (10 μl) was added, and the platelets were incubated with the anti‐integrin β_3_ mAb (1 μg/ml) overnight with rotation. The resulting complexes were then washed three times with TBST before they were analysed through immunoblotting as described previously.

### Platelet function analysis for whole blood

A Dade Behring PFA‐100 system (Dade Behring, Marburg, Germany) was used to analyse platelet function 15. Cartridges containing CADP‐coated membranes were preincubated with NCTD (0.15 and 0.3 μM) or the solvent control (0.1% DMSO) for 2 min. Whole blood aliquots of 0.8 ml were applied per cartridge, and then, the contents were exposed to high shear flow conditions (5000–6000/sec.). Closure time (CT) was defined as the time required for a platelet plug to occlude the aperture in the collagen membrane 15.

### Measurement of sodium fluorescein‐induced thrombus formation in mouse mesenteric microvessels

Male ICR mice (6 weeks) were anaesthetized using a mixture containing 75% air and 3% isoflurane maintained in 25% oxygen; their external jugular veins were then cannulated with a PE‐10 tube for administering the dye and drugs intravenously 16. Venules (30–40 μm) were irradiated at wavelengths of <520 nm to produce a microthrombus. Two NCTD doses (0.1 and 0.2 mg/kg) were administered 1 min. following sodium fluorescein (15 μg/kg) administration, and the time required for the thrombus to occlude the microvessel (occlusion time) was recorded. In this experiment, the method applied to the thrombogenic animal model conformed to the Guide for the Care and Use of Laboratory Animals (8th edition, 2011) and we received an affidavit of approval for the animal use protocol from Taipei Medical University (LAC‐2016‐0395).

### Measurement of bleeding time in mouse tail vein

The bleeding time was measured through transection of the tail of the mice. In brief, after 30 min. of administering either 0.2 mg/kg NCTD or 150 mg/kg aspirin intraperitoneally, we sharply cut the tail of the mice at 3 mm from the tip. The tails were immediately placed into a tube filled with saline at 37°C for measuring the bleeding time, which was recorded until the bleeding completely stopped.

### Statistical analysis

The experimental results are expressed as means ± standard errors of the means, along with the number of observations (*n*). Values of *n* refer to the number of experiments; each experiment was performed with different blood donors. The unpaired Student's *t*‐test was used to determine significant differences in the occlusion times of mice. The differences between the groups in other experiments were assessed using anova. When anova results indicated significant differences among group means, the groups were compared using the Student–Newman–Keuls method. A *P* value of *<*0.05 indicated statistical significance. Statistical analyses were performed with SAS (version 9.2; SAS Inc., Cary, NC, USA).

## Results

### Effects of NCTD on human platelet aggregation

As shown in Figure 1B, NCTD (0.1–0.5 μM) strongly and concentration‐dependently inhibited washed human platelet aggregation stimulated by either 1 μg/ml collagen or 0.01 IU/ml thrombin. At higher concentrations of 0.5–1.0 μM, NCTD exhibited similar inhibitory activity against platelet aggregation stimulated by 1 μM U46619, a prostaglandin endoperoxide or 60 μM AA. The 50% inhibitory concentrations (IC_50_) of NCTD for platelet aggregation induced by collagen, thrombin, U46619 and AA were approximately 0.25, 0.15, 0.9 and 0.8 μM, respectively; NCTD exhibited more potent inhibitory activity against thrombin stimulation than it did against other agonists (Fig. 1C). Moreover, aspirin (20, 50 and 100 μM) concentration‐dependently inhibited platelet aggregation stimulated by 1 μg/ml collagen, with an IC_50_ of approximately 50 μM (data not shown). Therefore, NCTD is approximately 200 times more potent than aspirin in inhibiting collagen‐stimulated platelet aggregation. The solvent control (0.1% DMSO) did not affect platelet aggregation significantly (Fig. 1B). In subsequent experiments, 0.01 IU/ml thrombin was used as the agonist for exploring the inhibitory mechanisms of platelet activation.

### Effects of NCTD on LDH release, ATP‐release reaction, surface P‐selectin expression and integrin α_IIb_β_3_ activation

**Figure 2:**
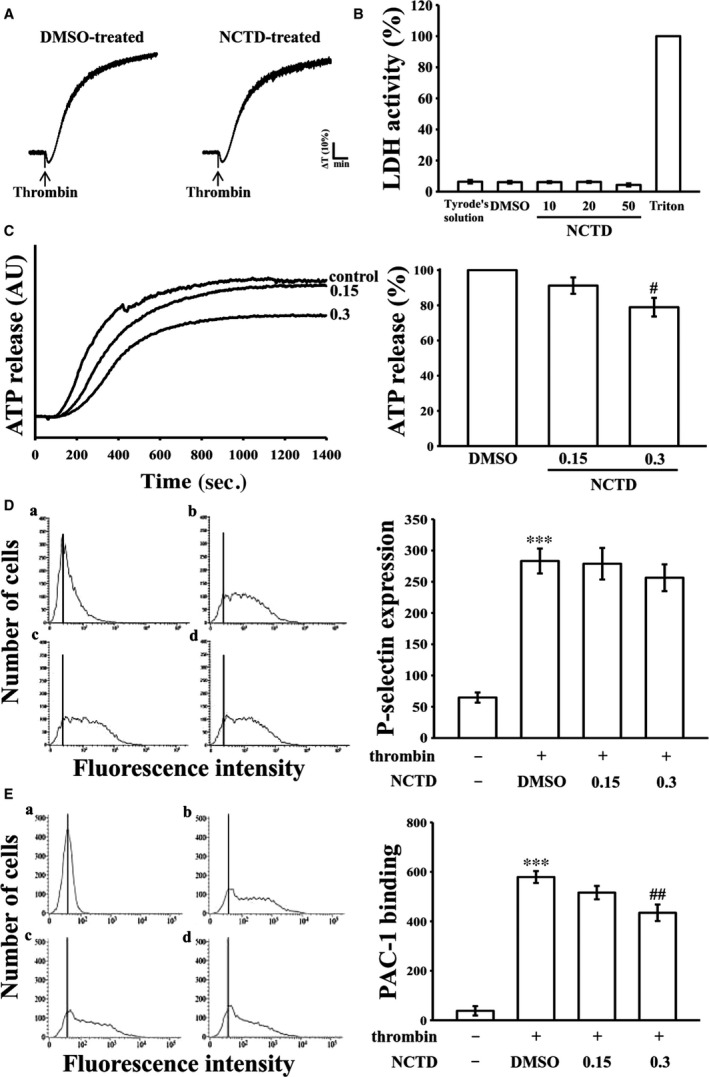
Effects of NCTD on cytotoxicity, lactate dehydrogenase (LDH) release, ATP‐release reaction, surface P‐selectin expression and integrin α_II__b_β_3_ activation in human platelets. (**A**) Washed platelets were preincubated with the solvent control (0.1% DMSO) or NCTD (5 μM) for 10 min. and subsequently washed two times with Tyrode solution; thrombin (0.01 U/ml) was then added to trigger platelet aggregation. (**B**) Washed human platelets (3.6 × 10^8^ cells/ml) were preincubated with NCTD (10, 20 and 50 μM) for 20 min., and a 10‐μl aliquot of the supernatant was deposited on a Fuji Dri‐Chem slide LDH‐PIII as described in ‘Methods’. (**C**) Moreover, washed platelets (3.6 × 10^8^ cells/ml) were preincubated with NCTD (0.15 and 0.3 μM) or the solvent control (0.1% DMSO), and 0.01 U/ml thrombin was then added to stimulate the ATP‐release reaction (AU; arbitrary unit). For other experiments (**D‐E**), resting platelets (a) or platelets (3.6 × 10^8^ cells/ml) were preincubated with the solvent control (b, 0.1% DMSO) or NCTD (c, 0.15; d, 0.3 μM) and the FITC‐conjugated anti‐P‐selectin mAb (2 μg/ml) or the PAC‐1 mAb (2 μg/ml) for 3 min. and then stimulated by thrombin (0.01 U/ml) for another 5 min. The suspensions were then assayed for fluorescein‐labelled platelets on a flow cytometer (FACScan system, Becton Dickinson). Profiles in (**A**) are representative of four independent experiments. Data in (**B‐E**) are presented as means ± standard errors of the means (*n *=* *4). ***P < 0.001, compared with the resting group; ^#^ *P < *0.05 and ^##^ *P < *0.01, compared with the 0.1% DMSO‐treated group.

The aggregation curves of platelets preincubated with 5 μM NCTD for 10 min. and subsequently washed two times with Tyrode's solution exhibited no significant differences from those of platelets preincubated with the solvent control (0.1% DMSO) under equivalent conditions (Fig. 2A), preliminarily indicating that the effects of NCTD on platelet aggregation are reversible and noncytotoxic. Furthermore, the LDH study revealed that NCTD (10, 20 and 50 μM) incubated with platelets for 20 min. did not significantly increase LDH activity or exert cytotoxic effects on platelets (Fig. 2B), demonstrating that NCTD does not affect platelet permeability or induce platelet cytolysis.

Platelet activation is associated with ATP release from dense granules and surface P‐selectin expression from α‐granules, thus causing ample platelet aggregation. In quiescent (resting) platelets, P‐selectin is located on the inner wall of α‐granules. Platelet activation results in ‘membrane flipping’, where the platelet releases α‐granules, exposing the inner walls of the granules on the outside of the cell 17. In this study, 0.15 and 0.3 μM NCTD only slightly reduced ATP release by approximately 8% and 21%, respectively; nevertheless, it had no effect on P‐selectin expression after 0.01 U/ml thrombin stimulation (Fig. 2C and D). Platelet aggregation is dependent on fibrinogen–integrin α_IIb_β_3_ binding; however, integrin α_IIb_β_3_ inactivation can lead to disaggregation of aggregated platelets 18. To further determine whether NCTD affects integrin α_IIb_β_3_ activation, the binding of the FITC‐conjugated PAC‐1 mAb specific for neoepitopes exposed on the activated form of integrin α_IIb_β_3_ was analysed through flow cytometry (Fig. 2E). NCTD (0.3 μM) considerably hindered integrin α_IIb_β_3_ activation stimulated by thrombin. This result indicates that the action mechanism of NCTD may be associated with its interference in fibrinogen–integrin α_IIb_β_3_ binding.

### NCTD restricts integrin α_IIb_β_3_‐mediated outside‐in signalling of cell adhesion and spreading as well as clot retraction

**Figure 3:**
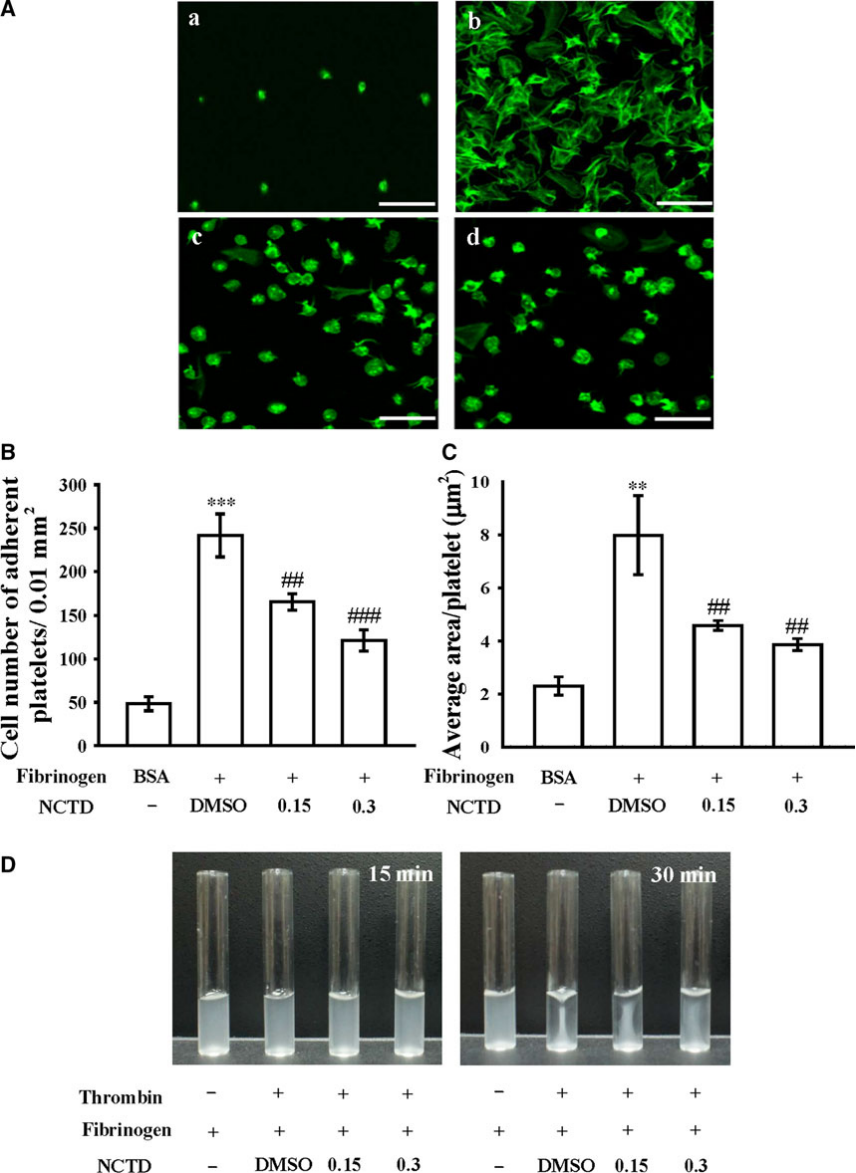
Controlling effects of NCTD on human platelet adhesion and spreading on immobilized fibrinogen as well as clot retraction. (**A**) Washed human platelets (3.0 × 10^8^ cells/ml) were allowed to spread on the (a) bovine serum albumin (BSA)‐ or (b‐d) fibrinogen‐coated surfaces at 37°C for 45 min. in the presence of the (b) solvent control (0.1% DMSO) or NCTD (c, 0.15; d, 0.3 μM; 5 min. at 37°C) and were then fixed with paraformaldehyde to stop spreading. Platelets were subsequently labelled with FITC‐conjugated phalloidin and photographed under a confocal microscope. (**B**) The number of adherent platelets per 0.01 mm^2^ and (**C**) the average spreading surface area of individual platelets in six sight views are plotted. (**D**) Washed platelets (3.6 × 10^8^ cells/ml) were suspended in Tyrode's solution containing 2 mg/ml fibrinogen and 1 mM CaCl_2_ with the solvent control (0.1% DMSO) or NCTD (0.15 and 0.3 μM). Clot retraction was initiated with thrombin (0.01 U/ml) at 37°C. Images were photographed at 15‐ and 30‐min. intervals using a digital camera. Profiles in (**A**) and (**D**) are representative of four similar experiments. Data in (**B**) and (**C**) are presented as means ± standard errors of the means (*n *=* *4). **P < 0.01 and ***P < 0.001, compared with the immobilized BSA group; ^##^ *P < *0.01 and ^###^ *P < *0.001, compared with the 0.1% DMSO‐treated group.

As shown in the Figure 3A*,* platelets staining with FITC‐conjugated phalloidin demonstrated that platelets adhered to immobilized fibrinogen were significantly more than immobilized BSA. In addition, NCTD‐treated platelets had lower adhesion to and spreading on immobilized fibrinogen than did 0.1% DMSO‐treated platelets (Fig. 3A). As shown in Figure 3B, control platelets were fixed to immobilized fibrinogen normally (241.7 ± 24.7 platelets/0.01 mm^2^; *n *=* *4), whereas NCTD‐treated platelets exhibited poorer adhesion to the fibrinogen‐coated surface (0.15 μM, 165.3 ± 9.1 platelets/0.01 mm^2^, *n *=* *4, *P *<* *0.01; 0.3 μM, 121.3 ± 12.2 platelets/0.01 mm^2^; *n *=* *4, *P *<* *0.001). Compared with 0.1% DMSO‐treated platelets (8.0 ± 1.5 μm^2^; *n *=* *4), the surface coverage of a single platelet treated with NCTD was reduced significantly (0.15 μM, 4.6 ± 0.2 μm^2^, *n *=* *4, *P *<* *0.01; 0.3 μM, 3.9 ± 0.2 μm^2^; *n *=* *4; *P *<* *0.01; Fig. 3C).

Clot retraction of fibrin polymers, the final step in thrombus formation, is essential in aggregate stabilization 19 and a paradigm of integrin α_IIb_β_3_ outside‐in signalling. A clot retraction assay was performed by adding thrombin into a solution containing fibrinogen in the presence of NCTD‐treated or 0.1% DMSO‐treated human platelets. As illustrated in Figure 3D, compared with that after 15‐min. incubation, clot retraction after 30‐min. incubation was more apparent for the 0.1% DMSO‐treated platelets, but it was substantially reduced for the 0.15 and 0.3 μM NCTD‐treated platelets. This result demonstrates that NCTD induces a deficit in the ability of platelets to mediate stable interactions with a fibrin matrix, reducing fibrin clot retraction.

### NCTD diminishes integrin α_IIb_β_3_‐mediated protein kinase activation

**Figure 4:**
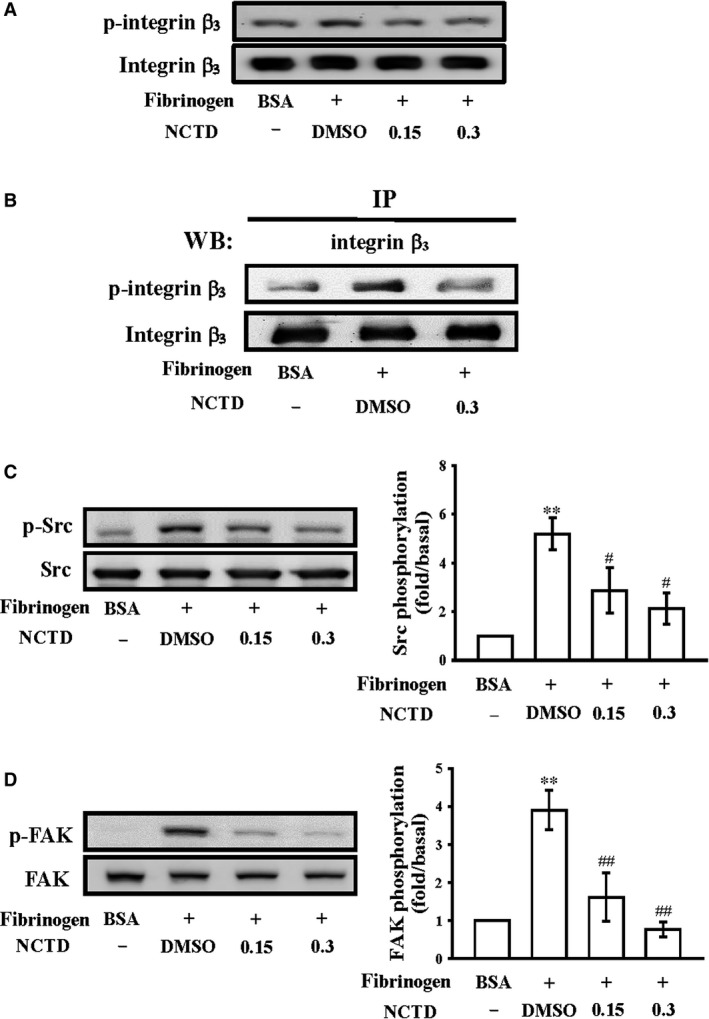
Effects of NCTD on phosphorylation of integrin β_3_, Src and FAK on a fibrinogen‐coated surface. Washed human platelets were preincubated with NCTD (0.15 and 0.3 μM) or the solvent control (0.1% DMSO) and subsequently activated by immobilized fibrinogen (100 μg/ml). Platelets were collected, and their subcellular extracts were analysed to determine the levels of (**A**) integrin β_3_, (**C**) Src and (**D**) FAK phosphorylation. (**B**) For immunoprecipitation study, washed platelets were preincubated with 0.3 μM NCTD or the solvent control (0.1% DMSO) for 3 min. and then allowed to spread on immobilized fibrinogen (100 μg/ml). Next, the platelets were lysed, Protein G Mag Sepharose Xtra beads (10 μl) were added with the anti‐integrin β_3_ mAb (1 μg/ml), and the platelets were incubated overnight with rotation for immunoblotting. The profiles in (**A**) and (**B**) represent four independent experiments; all data are presented as means ± standard errors of the means (*n = *4). **P < 0.01, compared with the immobilized bovine serum albumin (BSA)‐treated group; ^#^ *P < *0.05 and ^##^ *P < *0.01, compared with the immobilized fibrinogen‐treated group.

For further elucidating the mechanisms by which NCTD impairs integrin α_IIb_β_3_‐mediated outside‐in signalling, integrin β_3_ phosphorylation, a vital indicator of outside‐in signalling, was studied. First, we examined integrin β_3_ phosphorylation in platelets exposed to immobilized fibrinogen through an immunoblotting assay and observed that integrin β_3_ phosphorylation was significantly attenuated by NCTD (0.15 and 0.3 μM; Fig. 4A). Next, the proteins in the cellular extracts of the platelets were immunoprecipitated with the anti‐integrin β_3_ mAb, and the immunoprecipitates were analysed through immunoblotting with the anti‐phospho‐integrin β_3_ Ab; the results revealed that integrin β_3_ phosphorylation was significantly attenuated in the presence of 0.3 μM NCTD (Fig. 4B). In addition, biochemical studies on the lysates of platelets spreading on immobilized fibrinogen revealed that the pretreatment of platelets with NCTD inhibited immobilized fibrinogen‐induced phosphorylation of Src and FAK (Fig. 4C and D). Taken together, these data suggest that NCTD markedly impairs integrin α_IIb_β_3_‐mediated outside‐in protein phosphorylation.

### Inhibition of *ex vivo* and *in vivo* thrombus formation by NCTD

**Figure 5:**
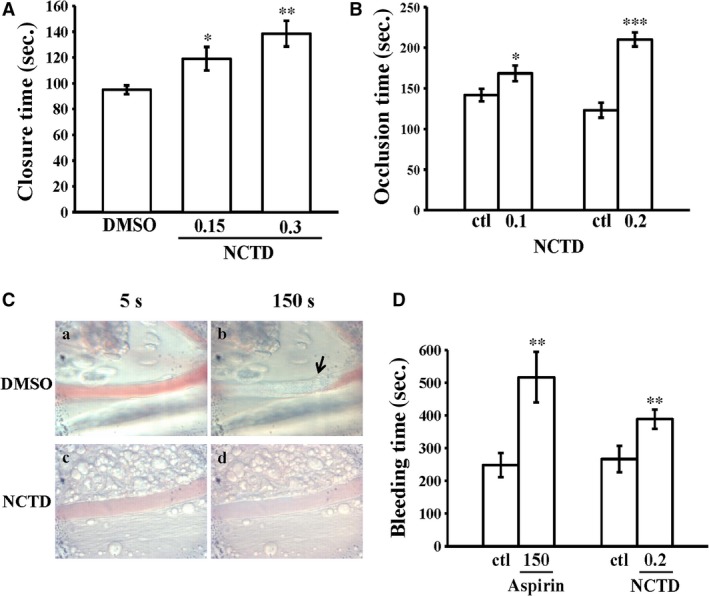
Protective effects of NCTD on closure time according to the analysis performed with the PFA‐100 system and thrombotic platelet plug formation in the mesenteric venules of mice as well as the bleeding time in mice tail vein. (**A**) Shear‐induced platelet plug formation in human whole blood was determined by recording the closure time of CADP‐coated membranes, as described in the ‘Materials and methods’. (**B**) For another study, mice were administered an intravenous bolus of the solvent control (ctl; 0.1% DMSO) or NCTD (0.1 and 0.2 mg/kg), and the mesenteric venules were irradiated to induce microthrombus formation (occlusion time). (**C**) Microscopic images (400 ×  magnification) of 0.1% DMSO‐treated controls (a and b) and the 0.2 mg/kg NCTD‐treated groups (c and d) were recorded at 5 sec. (a and c) and 150 sec. (b and d) after irradiation. The photographs are representative of six similar experiments. The arrow indicates platelet plug formation. **(D)** The bleeding time was measured through transection of the tail in mice after 30 min. of administering either 150 mg/kg aspirin or 0.2 mg/kg NCTD intraperitoneally. Data are presented as means ± standard errors of the means (**A**‐**B**,* n *=* *8; **D**,* n *=* *10). *P <* *0.05, **P <* *0.01 and ***P <* *0.001, compared with the 0.1% DMSO‐treated group.

Shear‐induced platelet plug formation in whole blood was analysed *ex vivo*. The PFA‐100 system was used to mimic the *in vivo* conditions of blood vessel injury. Platelets were exposed to a high shear rate, and the time required for platelet aggregation to occlude an aperture in a collagen‐coated membrane was recorded. The CT of the CADP‐coated membrane in whole blood treated with the solvent control (0.1% DMSO) was 95.0 ± 3.3 sec. (*n *=* *8; Fig. 5A). Treatment with 0.15 and 0.3 μM NCTD significantly increased this CT to 119.1 ± 9.1 sec. (*n *=* *8, *P *<* *0.05; Fig. 5A) and 138.6 ± 10.0 sec. (*n *=* *8, *P *<* *0.01; Fig. 5A), indicating that the adherence of platelets to collagen was prolonged under the flow conditions after NCTD treatment. Furthermore, we directly evaluated the antithrombotic activity of NCTD *in vivo*. The occlusion time in the mesenteric microvessels of mice pretreated with 15 μg/kg fluorescein sodium was approximately 120 sec. We administered NCTD at 0.1 or 0.2 mg/kg after pretreatment with fluorescein sodium; the resulting occlusion times were significantly prolonged after 0.1 and 0.2 mg/kg NCTD treatment compared with those after DMSO treatment (control, 141.8 ± 7.7 sec. *versus* 0.1 mg/kg NCTD, 168.5 ± 9.5 sec., *n *=* *8, *P *<* *0.05; control, 123.2 ± 9.3 sec. *versus* 0.2 mg/kg NCTD, 210.2 ± 8.7 sec., *n *=* *8, *P *<* *0.001; Fig. 5B). After irradiation, a thrombotic platelet plug was observed in the mesenteric microvessels at 150 sec., but not at 5 sec., in the DMSO‐treated group (Fig. 5Ca and b). On administration of 0.2 mg/kg NCTD, platelet plug formation was not observed at 5 or 150 sec. after irradiation (Fig. 5Cc and d). The observed blood flow rate in the control venules was lower than that in the NCTD‐treated venules because the platelet plug appeared only in the control venules at 150 sec. (Fig. 5Cb).

In our tail transection mouse model, after 30 min. of administering 150 mg/kg aspirin intraperitoneally, the bleeding time considerably increased from 248.5 ± 37.3 sec. (PBS‐treated control group; *n *=* *10) to 516.9 ± 77.5 sec. (*n *=* *10; *P *<* *0.01). The bleeding time of the 0.2 mg/kg NCTD‐treated mice was slightly longer than that of the solvent control (0.1% DMSO)‐treated mice (266.8 ± 40.0 sec. *versus* 388.7 ± 28.9 sec., *P *<* *0.01; *n *=* *10) (Fig. 5D).

## Discussion

NCTD is a potent drug capable of chemoprevention and tumour inhibition; therefore, it is suitable for clinical anticancer applications. The beneficial effects of NCTD include overcoming multidrug resistance and radiation sensitization; thus, NCTD is considered one of the most promising anticancer agents in China 11. Notably, our results, for the first time, reveal that, in addition to its antitumour activity, NCTD exhibits highly potent antiplatelet activity (IC_50_, 0.15 μM) *ex vivo* and successfully inhibits arterial thrombogenesis (0.1 mg/kg) *in vivo*. Platelets are activated by various physiological stimuli (*e.g*. collagen, thrombin and AA); these stimuli are considered to exert their effects by interacting with specific receptors on the platelet membrane. Aspirin has been clinically used for treating and preventing cardiovascular diseases (CVDs). Our current results demonstrate that, under identical conditions, NCTD has antiplatelet activity that is over 200 times more potent than that of aspirin, indicating that NCTD has potential for clinical application in CVD treatment.

NCTD inhibited platelet aggregation stimulation by agonists (*i.e*. collagen, thrombin, U46619 and AA) within a limited concentration range (0.1–1 μM), indicating that NCTD was ineffective against individual receptors of these agonists. Thus, NCTD probably acts through a common signal cascade against stimulated platelets. A major component of the platelet aggregation response is fibrinogen–integrin α_IIb_β_3_ binding on activated platelets. Integrin α_IIb_β_3_ undergoes conformational changes upon activation by agonists, such as collagen and thrombin, developing a specific ligand‐binding site for fibrinogen, von Willebrand factor, fibronectin and vitronectin 2. This conformational change is crucial for platelet–platelet adhesion because the ligand‐bound integrin α_IIb_β_3_ promotes platelet aggregation. Triflavin, an Arg‐Gly‐Aso‐containing disintegrin, acts as a specific antagonist for integrin α_IIb_β_3_ and directly interferes with the fibrinogen–integrin α_IIb_β_3_ interaction 20. Triflavin inhibited platelet aggregation stimulated by various agonists (*i.e*. collagen, thrombin and U46619), without affecting the ATP‐release reaction and the initial shape change in platelet aggregation 21. Our results are consistent with studies of triflavin, in that NCTD inhibited platelet aggregation stimulated by all studied agonists without affecting initial shape change and P‐selectin expression in addition to only slightly reducing ATP‐release reaction. In addition, PAC‐1 reacts with the activation‐induced conformational epitope of integrin α_IIb_β_3_ 22. In this study, NCTD noticeably reduced the binding of PAC‐1 to integrin α_IIb_β_3_ in thrombin‐activated platelets. Furthermore, integrin β_3_ phosphorylation stimulated by immobilized fibrinogen was alleviated by NCTD; therefore, the antiplatelet activity of NCTD possibly involves its direct interference with fibrinogen–integrin α_IIb_β_3_ binding to subsequently block integrin α_IIb_β_3_‐mediated outside‐in signalling.

Moreover, platelet‐mediated clot retraction is mediated by integrin α_IIb_β_3_. In turn, different pathways of protein phosphorylation regulate integrin α_IIb_β_3_ activation through inside‐out mechanisms and posterior outside‐in signalling 23. Integrin α_IIb_β_3_‐mediated signalling actually begins immediately after a fibrinogen molecule binds to the integrin; this outside‐in signalling results in tyrosine phosphorylation of numerous proteins, such as the Src family kinases (SFK; *e.g*. Src, Lyn and Fyn), FAK and the cytoplasmic tail of integrin β_3_ at Tyr^759^, a process dependent on outside‐in signalling and cytoskeleton reorganization 2. The critical role of integrin β_3_ at Tyr^759^ in platelet physiology was demonstrated *in vivo*, and its mutation leads to bleeding disorder and strongly affects clot retraction responses *in vitro* 24. FAK, a cytoplasmic tyrosine kinase located at focal adhesion points, plays a vital role in cytoskeleton regulation and integrin α_IIb_β_3_ activity 25. Platelet adhesion to immobilized fibrinogen requires FAK activation through integrin α_IIb_β_3_, and in turn, activation of FAK requires autophosphorylation 26. In this study, NCTD noticeably abolished platelet adhesion and spreading and clot retraction as well as phosphorylation of integrin β_3_, Src and FAK on immobilized fibrinogen in the absence of platelet agonists. Thus, NCTD potentially acts on integrin α_IIb_β_3_ and blocks integrin α_IIb_β_3_‐mediated outside‐in signalling.

After vascular endothelial cell injury, exposure to subendothelial collagen is the major trigger that initiates platelet adhesion and aggregation at the injury site, followed by arterial thrombus formation. The PFA‐100 system records the time required for platelet aggregation to occlude an aperture in a collagen‐coated membrane. Platelet adhesion to collagen depends on the flow conditions. In this study, platelets prolonged adhesion to collagen under the flow conditions. In a thrombosis study 13, mesenteric venules were continuously irradiated by fluorescein sodium throughout the experimental period, leading to strong damage to the endothelial cells. Here, NCTD significantly prolonged both CTs and occlusion times; these effects may be mediated, at least partly, by the inhibition of platelet activation. In addition, we used the tail transection mouse model to examine the effects of NCTD on bleeding time *in vivo*. Although aspirin is the most effective antiplatelet drug prescribed for preventing or treating cardiovascular and cerebrovascular diseases, it causes unwanted prolongation of bleeding time. In tail transection mouse, the bleeding time of the NCTD‐treated mice was slightly longer than that of the solvent control, indicating that the slight prolongation of bleeding time may be caused by the antiplatelet activity of NCTD, at least partly. Furthermore, Wei *et al*. 27 have reported that healthy human volunteers were orally administered 10 mg NCTD tablets, and it was found to absorb quickly with an absorption half‐life of 0.81 hr, time to peak concentration of 2.00 hr, maximum serum concentration of 33.24 ng/ml, elimination half‐life of 0.81 hr and mean retention time of 6.64 hrs. In conclusion, the findings of this study reveal that NCTD has a novel and alternative role in inhibiting platelet activation through directly interfering with fibrinogen–integrin α_IIb_β_3_ binding and subsequently blocking integrin α_IIb_β_3_‐mediated outside‐in signalling. However, the involvement of other mechanisms, which are yet to be identified, in NCTD‐mediated inhibition of platelet activation requires investigation. Nevertheless, NCTD can be a chemotherapeutic agent for cancer treatment and exhibits potent antiplatelet activity for treating thromboembolic disorders.

## Authors’ contributions

C.H.H. performed the research and wrote the manuscript; K.H.L., W.J.L. and D.S.C. performed the research and some experiments; P.G., T.J. and N.C.C. performed some experiments and analysed the data; J.R.S. conceived the study and designed the research. All authors have read and approved the final manuscript.

## Conflict of interest

The authors confirm that there is no conflict of interests.

## References

[1] Jayakumar T , Yang CH , Geraldine P , *et al* The pharmacodynamics of antiplatelet compounds in thrombosis treatment. Expert Opin Drug Metab Toxicol. 2016; 12: 2142–2152. 10.1080/17425255.2016.1176141 27055051

[2] Payrastre B , Missy K , Trumel C , *et al* The integrin α_IIb_/β_3_ in human platelet signal transduction. Biochem Pharmacol. 2000; 60: 1069–74. 11007943 10.1016/s0006-2952(00)00417-2

[3] Tesfamariam B . Involvement of platelets in tumor cell metastasis. Pharmacol Ther. 2016; 157: 112–9. 26615781 10.1016/j.pharmthera.2015.11.005

[4] Nierodzik ML , Klepfish A , Karpatkin S . Role of platelets, thrombin, integrin IIb/IIIa, fibronectin and von Willebrand factor on tumor adhesion *in vitro* and metastasis *in vivo* . Thromb Haemost. 1995; 74: 282–90. 8578473

[5] Bakewell SJ , Nestor P , Prasad S , *et al* Platelet and osteoclast beta3 integrins are critical for bone metastasis. Proc Natl Acad Sci USA. 2003; 100: 14205–10. 14612570 10.1073/pnas.2234372100 PMC283570

[6] Nierodzik ML , Karpatkin S . Thrombin induces tumor growth, metastasis, and angiogenesis: evidence for a thrombin‐regulated dormant tumor phenotype. Cancer Cell. 2006; 10: 355–62. 17097558 10.1016/j.ccr.2006.10.002

[7] Ekambaram P , Lambiv W , Cazzolli R , *et al* The thromboxane synthase and receptor signaling pathway in cancer: an emerging paradigm in cancer progression and metastasis. Cancer Metastasis Rev. 2011; 30: 397–408. 22037941 10.1007/s10555-011-9297-9 PMC4175445

[8] Medina C , Jurasz P , Santos‐Martinez MJ , *et al* Platelet aggregation induced by Caco‐2 cells: regulation by matrix metalloproteinase‐2 and adenosine diphosphate. J Pharmacol Exp Ther. 2006; 317: 739–45. 16424148 10.1124/jpet.105.098384

[9] Wang GS . Medical uses of mylabris in ancient China and recent studies. J Ethnopharmacol. 1989; 26: 147–62. 2689797 10.1016/0378-8741(89)90062-7

[10] Deng LP , Dong J , Cai H , *et al* Cantharidin as an antitumor agent: a retrospective review. Curr Med Chem. 2013; 20: 159–66. 23210849 10.2174/092986713804806711

[11] Hsieh CH , Chao KS , Liao HF , *et al* Norcantharidin, derivative of cantharidin, for cancer stem cells. Evid Based Complement Alternat Med. 2013; 2013: 838651. 24073010 10.1155/2013/838651 PMC3773992

[12] Shou LM , Zhang QY , Li W , *et al* Cantharidin and norcantharidin inhibit the ability of MCF‐7 cells to adhere to platelets *via* protein kinase C pathway‐dependent downregulation of α_2_ integrin. Oncol Rep. 2013; 30: 1059–66. 23835679 10.3892/or.2013.2601 PMC3783059

[13] Sheu JR , Lee CR , Lin CH , *et al* Mechanisms involved in the antiplatelet activity of *Staphylococcus aureus* lipoteichoic acid in human platelets. Thromb Haemost. 2000; 83: 777–84. 10823277

[14] Osdoit S , Rosa JP . Fibrin clot retraction by human platelets correlates with alpha(IIb)beta(3) integrin‐dependent protein tyrosine dephosphorylation. J Biol Chem. 2001; 276: 6703–10. 11084040 10.1074/jbc.M008945200

[15] Jilma B . Platelet function analyzer (PFA‐100): a tool to quantify congenital or acquired platelet dysfunction. J Lab Clin Med. 2001; 138: 152–63. 11528368 10.1067/mlc.2001.117406

[16] Hsiao G , Lin KH , Chang Y , *et al* Protective mechanisms of inosine in platelet activation and cerebral ischemic damage. Arterioscler Thromb Vasc Biol. 2005; 25: 1998–2004. 15976325 10.1161/01.ATV.0000174798.25085.d6

[17] Harrison P , Cramer EM . Platelet α‐granules. Blood Rev. 1993; 7: 52–62. 8467233 10.1016/0268-960x(93)90024-x

[18] Cosemans JM , Iserbyt BF , Deckmyn H , *et al* Multiple ways to switch platelet integrins on and off. J Thromb Haemost. 2008; 6: 1253–61. 18513212 10.1111/j.1538-7836.2008.03041.x

[19] Shattil SJ . The beta3 integrin cytoplasmic tail: protein scaffold and control freak. J Thromb Haemost. 2009; 7: 210–3. 19630802 10.1111/j.1538-7836.2009.03397.x

[20] Sheu JR , Hung WC , Wu CH , *et al* Reduction in lipopolysaccharide‐induced thrombocytopenia by triflavin in a rat model of septicemia. Circulation. 1999; 99: 3056–62. 10368125 10.1161/01.cir.99.23.3056

[21] Huang TF , Sheu JR , Teng CM . A potent antiplatelet peptide, triflavin, form *Trimeresurus flavoviridis* snake venom. Biochem J. 1991; 277: 351–7. 1859363 10.1042/bj2770351 PMC1151241

[22] Shattil SJ , Cunningham M , Hoxie JA . Detection of activated platelets in whole blood using activation‐dependent monoclonal antibodies and flow cytometry. Blood. 1987; 70: 307–15. 3297204

[23] Shattil SJ . Integrins and Src: dynamic duo of adhesion signaling. Trends Cell Biol. 2005; 15: 399–403. 16005629 10.1016/j.tcb.2005.06.005

[24] Law DA , DeGuzman FR , Heiser P , *et al* Integrin cytoplasmic tyrosine motif is required for outside‐in α_IIb_β_3_ signalling and platelet function. Nature. 1999; 401: 808–11. 10548108 10.1038/44599

[25] Schaller MD . Cellular functions of FAK kinases: insight into molecular mechanisms and novel functions. J Cell Sci. 2010; 123: 1007–13. 20332118 10.1242/jcs.045112

[26] Ji P , Haimovich B . Integrin alpha IIb beta 3‐mediated pp125FAK phosphorylation and platelet spreading on fibrinogen are regulated by PI 3‐kinase. Biochim Biophys Acta. 1999; 1448: 543–52. 9990307 10.1016/s0167-4889(98)00160-8

[27] Wei CM , Zhang R , Wang BJ , *et al* Determination and pharmacokinetic study of norcantharidin in human serum by HPLC‐MS/MS method. Biomed Chromatogr. 2008; 22: 44–9. 17849504 10.1002/bmc.892

